# A Detailed Overview of SARS-CoV-2 Omicron: Its Sub-Variants, Mutations and Pathophysiology, Clinical Characteristics, Immunological Landscape, Immune Escape, and Therapies

**DOI:** 10.3390/v15010167

**Published:** 2023-01-05

**Authors:** Srijan Chatterjee, Manojit Bhattacharya, Sagnik Nag, Kuldeep Dhama, Chiranjib Chakraborty

**Affiliations:** 1 Department of Biotechnology, School of Life Science and Biotechnology, Adamas University, Kolkata 700126, West Bengal, India; 2 Department of Zoology, Fakir Mohan University, Vyasa Vihar, Balasore 756020, Odisha, India; 3 Department of Biotechnology, School of Biosciences & Technology, Vellore Institute of Technology (VIT), Vellore 632014, Tamil Nadu, India; 4 Division of Pathology, ICAR-Indian Veterinary Research Institute, Izatnagar, Bareilly 243122, Uttar Pradesh, India

**Keywords:** Omicron: sub-lineages, transmission and infection, disease severity

## Abstract

The COVID-19 pandemic has created significant concern for everyone. Recent data from many worldwide reports suggest that most infections are caused by the Omicron variant and its sub-lineages, dominating all the previously emerged variants. The numerous mutations in Omicron’s viral genome and its sub-lineages attribute it a larger amount of viral fitness, owing to the alteration of the transmission and pathophysiology of the virus. With a rapid change to the viral structure, Omicron and its sub-variants, namely BA.1, BA.2, BA.3, BA.4, and BA.5, dominate the community with an ability to escape the neutralization efficiency induced by prior vaccination or infections. Similarly, several recombinant sub-variants of Omicron, namely XBB, XBD, and XBF, etc., have emerged, which a better understanding. This review mainly entails the changes to Omicron and its sub-lineages due to it having a higher number of mutations. The binding affinity, cellular entry, disease severity, infection rates, and most importantly, the immune evading potential of them are discussed in this review. A comparative analysis of the Delta variant and the other dominating variants that evolved before Omicron gives the readers an in-depth understanding of the landscape of Omicron’s transmission and infection. Furthermore, this review discusses the range of neutralization abilities possessed by several approved antiviral therapeutic molecules and neutralizing antibodies which are functional against Omicron and its sub-variants. The rapid evolution of the sub-variants is causing infections, but the broader aspect of their transmission and neutralization has not been explored. Thus, the scientific community should adopt an elucidative approach to obtain a clear idea about the recently emerged sub-variants, including the recombinant variants, so that effective neutralization with vaccines and drugs can be achieved. This, in turn, will lead to a drop in the number of cases and, finally, an end to the pandemic.

## 1. Introduction

The SARS-CoV-2 virus is highly infectious, and it was the causative agent of the outbreak of the COVID disease in 2019. The WHO declared it to be a global pandemic [1,2]. More than 480 million cases have already been reported worldwide, with there having been over 6 million deaths since late 2019 [3]. Most of the infected people develop mild-to-moderate symptoms such as a cough, fever, the loss of smell and taste, a headache, sore throat, diarrhea, body aches, and tiredness. The virus kept evolving, and APOBEC-induced mutations contributed to SARS-CoV-2’s evolution and fitness, and different variants were identified during the pandemic [4,5]. The variants were classified as variants under monitoring (VUMs), variants of concern (VOCs), and variants of interest (VOIs). These variants are Alpha (B.1.1.7), Beta (B.1.351), Gamma (P.1), Delta (B.1.617.2), and a novel or new variant, Omicron (B.1.1.529), which has a much faster infection rate than the other four variants do [6]. A new variant’s threat depends on three main factors and their interactions. Those factors are its transmissibility, severity compared to other strain (fewer ICU hospitalizations), and immune evasion. The variants have evolved by multiple mutations in inconsistent combinations, mainly in the spike protein (S1 and S2 subunits) of the virus, which helps to initiate the coronavirus’ life cycle. The important mutations that play a crucial role in the infectivity of the variants are Alpha, with an N501Y mutation in the RBD, Beta with N501Y, K417N, and E484K mutations in the RBD regions, Gamma with N501Y, K417T, and E484K mutations in the RBD regions, Delta with T478K, L452R mutations in the RBD regions, and Omicron with S371L, G339D, S375F, S373P, K417N, N440K, S477N, G446S, E484A, T478K, Q493K, Q498R, G496S, N501Y, and Y505H mutations in the RBD regions [7,8,9,10]. The symptoms of the Omicron infection are less dangerous than those of the other strains, but it is more transmissible and less susceptible to vaccines, even though the mortality rate is lower than those of other strains [11,12,13].

**Figure 1:**
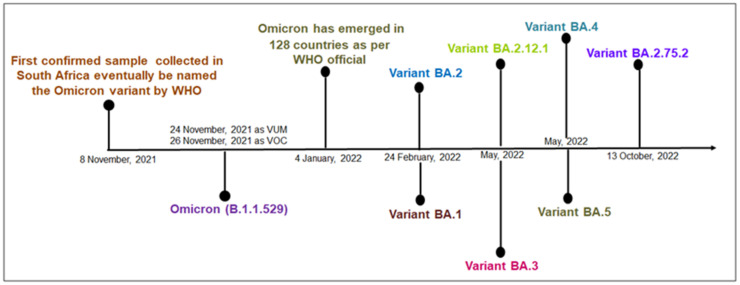
A timeline describes the origin of SARS-CoV-2 Omicron and different times of origin of Omicron’s sub-variants.

**Figure 2:**
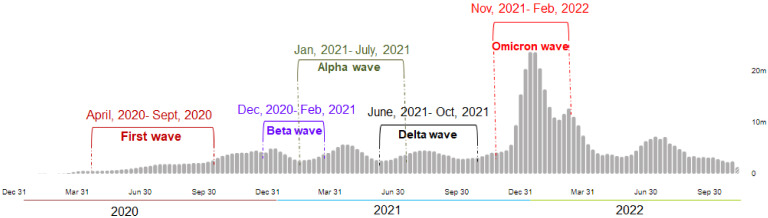
The new wave generated due to high infection worldwide due to Omicron’s infection. The new wave is called the Omicron wave. The peak of the Omicron wave is very high compared to the other waves, such as the Alpha wave and the Beta wave, etc.

Omicron was first spotted in South Africa and Botswana in November 2021 [14] (Figure 1). More than 130 million cases including 500,000 deaths have been reported worldwide since Omicron was declared as a VOC by the WHO, leading to a 44% rise in the average number of COVID-19 cases. The basic reproduction number (R0) of the Omicron variant was reported as being 8.2, showing an increased rate of transmissibility compared to that of the Delta variant [15]. The R0 of the Delta variant was observed as being between 3.2 and 8 [16]. It is estimated that Omicron infects 3–6 times more people as compared to Delta during a given time frame [17]. During the period when Omicron predominated, the rate of weekly hospitalization per 100,000 adults peaked at 38.4 compared to that of the Delta variant, which was 15.5 during predominant period in the United States. The Omicron variant has generated a new wave, evidenced by high infection rates worldwide [18]. The new wave is called the Omicron wave. The peak of the Omicron wave is very high compared to the waves of the variants such as the Alpha and Beta, etc., (Figure 2). Due to mutations in the genomic region of the variants, several subtypes have emerged over time, which are named sub-variants. The noteworthy evolution of the genomic regions of the Omicron variants has resulted in the emergence of several sub-lineages or sub-variants, which are denoted as BA.1, BA.2, BA.3, BA.4, BA.5, and recombinant BA.1/BA.2 [19]. Several researchers have tried to understand the Omicron sub-variants in more detail [13,20]. The BA.1 sub-lineage was the most prevalent globally, but BA.2 progressively replaced BA.1 in numerous countries, and the transmissibility of BA.3 is very restricted, with it having the lowest number of cases [16] (Figure 1). The other two new lineages detected in South Africa during January and February 2022 were BA.4 and BA.5, respectively [21] (Figure 1). These two lineages were predominant in the 5th wave of COVID-19 pandemic that was initiated in South Africa, and it replaced BA.2, as more than 50% of the cases were due to the dominance of BA.4 (35%) and BA.5 (20%) [9]. Omicron has more mutations than any other variant does. Omicron’s mutation helps it to bind more strongly with the ACE2 host cell receptors than the other reported variants can [22]. It also evades most of the virus-blocking antibodies or the ‘neutralizing’ antibodies (but not all of them) produced by vaccinated individuals or individuals infected with the other variants [23,24,25].

The main hindrance in the generation of antibodies and the development of suitable vaccines and therapeutic agents is due to the number of escape mutations generated in the genome of the SARS-CoV-2 virus and the sudden appearance of new strains. It is resistant to some existing vaccines and therapeutic agents. Studying the conformational dynamics of different antibody neutralization escape mutants is thus very important [26,27,28,29]. Additionally, understanding the antibodies targeted to different regions (S1/S2) of the spike protein which inhibit viral entry is essential for designing new antibodies. It can target the spike protein’s local dynamics, decreasing the efficacy of viral inhibition by the antibodies. Generating synthetic vaccines depending on the conformational dynamics of the variants will also be economical and they will be easy to update as they contain parts that are easily replaceable to act against the new strains of the pathogens.

In the present article, we have enlisted and summarized the different mutations of the Omicron variant and its sub-variants, along with the pathophysiology, clinical characteristics, and associated disease severity. Subsequently, we have highlighted the infection, reinfection, and transmissibility of the Omicron variant and its sub-variants, including the specific immunological features inside the host cells. Furthermore, a particular emphasis is also placed on the proposed small molecules and antibody-based therapeutics against Omicron and its sub-variants. This evidenced-based review will support future researchers in formulating appropriate strategies to resist the infections caused by Omicron and its sub-variants in the future.

## 2. Sub-Variants of Omicron Variant

**Figure 3:**
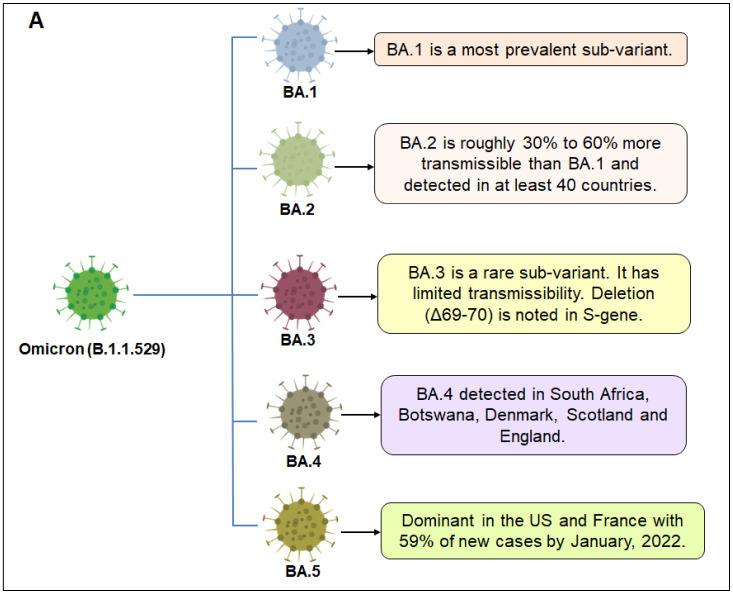

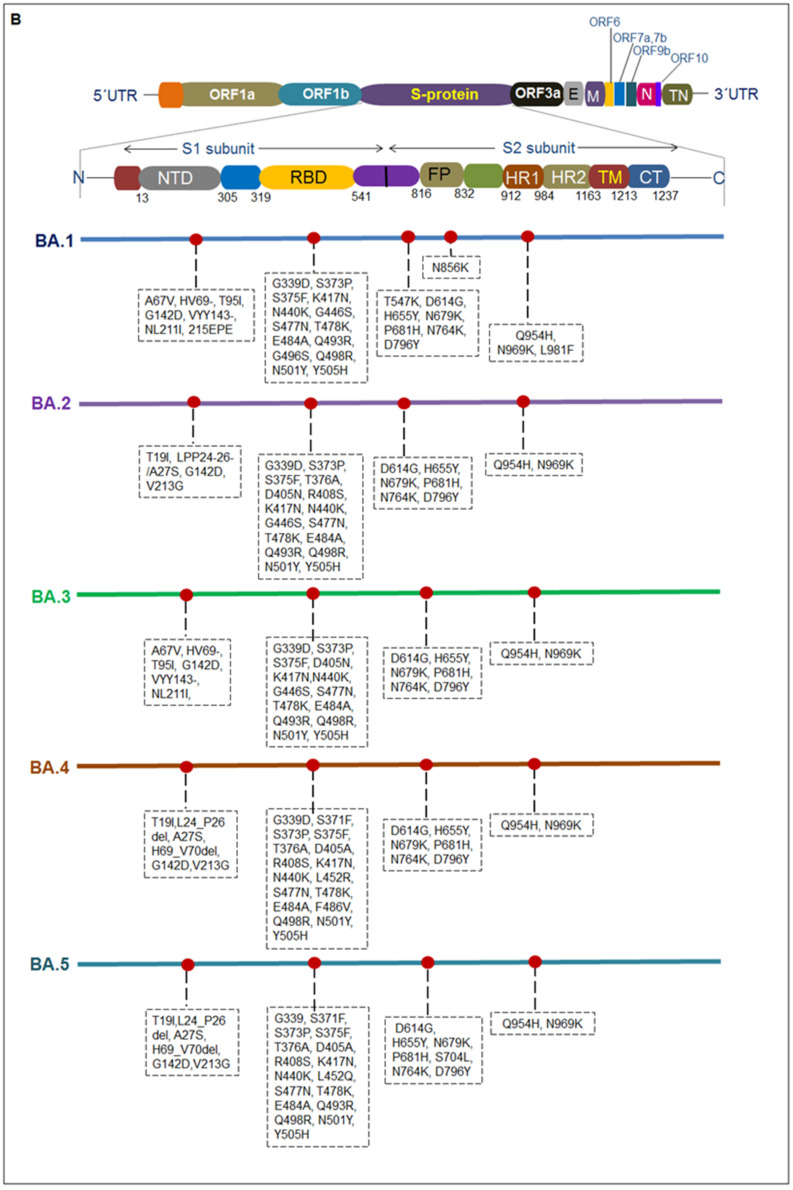
The figure illustrates different sub-variants of Omicron and their significant mutations in S-glycoprotein. (**A**) The figure describes different sub-variants of Omicron and their features. It describes the features of BA.1, BA.2, BA.3, BA.4, and BA.5. (**B**) The figure illustrates different mutations in S-glycoprotein of different sub-variants of Omicron. It describes the S-glycoprotein mutations of BA.1, BA.2, BA.3, BA.4, and BA.5.

Omicron has many mutations in its viral genome. According to the reports published on April 2022 by the WHO, five sub-variants of the Omicron variant have been detected. They are BA.1, BA.2, BA.3, BA.4, and BA.5. These mutations have been prevalent worldwide in different quantities (Figure 3A) [30]. Kumar et al. applied an elucidated approach using certain computational tools to provide insight regarding the pathogenicity and infectivity of the S-glycoprotein of BA.1 and the corresponding sub-lineages, BA.1.1, BA.2 and BA.3. The BA.1 sub-lineage shares 39 substitutions in the genome, followed by 40 mutational changes residing in the genome of BA.1.1 [31]. On the contrary, the BA.2 and BA.3 variants also share 31 and 34 mutations in their genome. Significantly, 21 mutations are prevalent in all of the evolved sub-lineages of the Omicron variant. Furthermore, 11 usual mutational changes have been deciphered in the RBD of the Omicron variant and the evolved sub-variants. The T95I, V213R, Y505H, N856K, N786K, and N211I mutations residing in Omicron and its sub-variants are highly pathogenic (Figure 3B) [31]. Reports have highlighted that no substantial mutations have been observed in the BA.3 variant’s spike glycoprotein. The mutations in the spike protein of BA.1 and BA.2 are only observable in the lately emerged BA.3 sub-lineage [32]. The data retrieved from the following website, https://outbreak.info/ (accessed on 6 August, 2022), provide the ratio, which shows the dominance of the Omicron sub-variants worldwide. The first sub-lineage, BA.1, accounts for 5% of the total number of cases in 161 countries, which is followed by the BA.1.1 variant, possessing 17% of the cases in the same countries. A steeper rise in the number of cases was prevalent for the BA.2 sub-variant, i.e., 9% of the reported cases across 163 different countries, with an extremely low prevalence of BA.3 cases until May 2022 [31]. The most alarming insight is the ability of these variants to escape the immune system and decrease the neutralization efficiency of vaccines. It was observed that the BA.1 sub-variant is more transmissible than the previously emerged Delta variant is, but the infected people rarely require hospital support. Owing to the presence of the H78Y mutation, the BA.2 sub-variant is more severe than the BA.1 sub-variant is [33]. The latest transmission rates account for the BA.3 sub-lineage because they lack six mutations in the genome, namely, L981F, G496S, ins214EPE, N856K, T547K, and S371L [32].

In early 2022, scientists also found two more sub-lineages of the Omicron variant from South African, namely, BA.4 and BA.5. After its report in South Africa, this sub-variant emerged in many areas across the globe. At the end of 2021, the BA.1 variant replaced the Delta one, and it was considered to be the leading causative agent of the fourth wave. Similarly, this BA.1 sub-variant was again replaced by BA.2, manifesting its dominance by April 2022 [33,34]. These two newly emerged sub-variants are the key factor responsible for the fifth wave of COVID-19. These variants are replacing all of the previously emerged sub-lineages of Omicron. The spike proteins of these recently evolved sub-lineages are somewhat similar. The BA.4 and BA.5 sub-variants possess certain extra mutations in the viral genome, with a synonymous substitution that is similar to the B.1.429 SARS-CoV-2 variant, which was also seen in BA.2 [35]. These two sub-lineages also show potent activities in evading the host’s immune system. However, the appropriate information regarding the hospitalization rates of the victims of BA.4 or BA.5 has remained unexplored. One of the recently evolved sub-variants, BA.2.12.1, exhibits a key feature of invalidating the antibodies present in the host due to vaccination or prior infection with the Omicron variant [36]. In a nutshell, it can be concluded that the BA.4 and BA.5 sub-lineages, along with the BA.2.12.1 one, are more robust and can evade the host’s humoral immunity [37]. 

Apart from these sub-lineages, several hybrid combinations of these sub-variants are also prevailing in the community, such as the XD, XE, and XF ones. The XE variant is a combination of the BA.1 and BA.2 sub-lineages, which has significantly worse effects, raising severe concerns for global health amidst the pandemic [38,39,40,41]. Similarly, XD is considered to be a recombinant form of the BA.1 and Delta variants, while the XF one is a product of the BA.1 sub-lineage and the Delta strains isolated from the United Kingdom. The WHO has termed the XE variant as “stealth Omicron,” possessing a ten times higher infectivity rate than the BA.2 sub-lineage does [41]. Scientists are worried about the severity of the infections caused by these recombinant variants. A closer look at the mutation profile of the XE variants revealed three new mutations, namely, C14599T, V1069I, and C3241T, which were not reported in the BA.1 and BA.2 sub-variants. The XD hybrid was first one to be reported from France. It contains a new mutation in the nsp2 gene, i.e., E172D, whereas the XF variant possesses a breakpoint at the end of the nsp3 gene, which is not common in the other sub-variants [40].

Recently, scientists noted that numerous other recombinant sub-variants have evolved during the post-Omicron period, such as the XBB, XBD, and XBF ones, etc., whose pathophysiology are yet to be studied [42].

## 3. Different Mutations and Pathophysiology Condition

**Figure 4:**
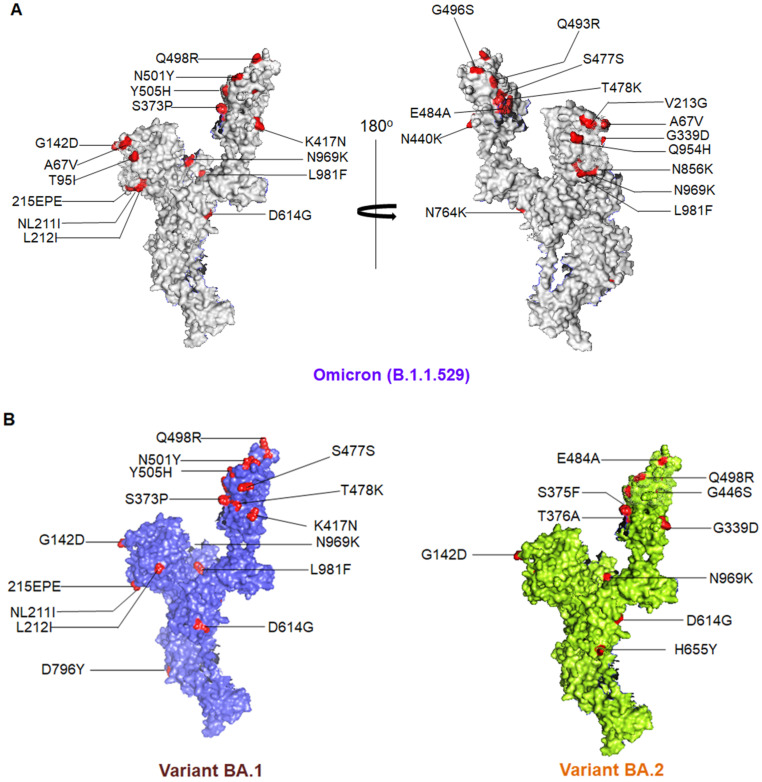
The figure shows different significant mutations in a 3D model of the S-glycoprotein of Omicron and its different sub-variants. (**A**) The figure shows different significant mutations in a 3D model of the S-glycoprotein of Omicron. (**B**) The figure shows different significant mutations in a 3D model of the S-glycoprotein of Omicron’s sub-variants, BA.1 and BA.2.

Omicron has more than 50 known mutations, 32 of which are in the spike protein rather than the wild-type one [43]. The Delta strain, in comparison, has nine mutations in the spike protein itself and thirteen mutations in the added regions. Out of the fifty mutations, twenty-six of them are unique to Omicron, and it also has ten mutations that are unique to Delta and six mutations that are unique to the Beta strain [44]. The mutations that the Omicron lineage possesses are ORF1a-6 substitutions at K856R, A2710T, L2084I, P3395H, T3255I, and I3758V, two deletions at positions 2083 and 3674–3676, ORF1b-2 substitutions at P314L and I1566V, deletions at positions 27, 28, and 29, and a P10S substitution at ORF9b. The mutations in the structural proteins are an envelope (E)-T9I substitution, membrane (M)- D3G, Q19E, and A63T substitutions, a nucleocapsid (N)- a three residue deletion, and three residue substitutions. The significant spike (S) mutations are A67V, T95I, L212I, Y145D, G339D, S373P, S371L, K417N, S375F, N440K, G446S, S477N, E484A, T478K, Q493R, N856K, Q498R, G496S, N501Y, Y505H, T547K, Q954H, P681H, D614G, H655Y, N764K, N679K, N969K, and D796Y, etc., (Figure 4A). Some other mutations are an L981F substitution, H69/V70, G142/V143/Y144, and N211 deletions, and an insertion of amino acid EPE at position 214 [3,45].

The BA.2 lineage consists of 57 mutations, of which 31 are in the S protein, and the N-terminus is exclusively different from that of the BA.1 lineage, whereas 12 mutations are common in both the BA.1 and BA.2 lineages in the RBD region, which are G339D, K417N, S373P, S375F, T478K, N440K, S477N, E484A, Q498R, N501Y, Q493R, and Y505H (Figure 4B). G446S, S371L, and G496S are unique to the BA.1 lineage, and R346K is found in a member of the BA.1 lineage, namely, BA.1.1.

Omicron and its variants have several unique mutations in the RBD region. The RBD mutations might control the functionality of that specific RBD region. The BA.2 lineage has two unique mutations in RBD, R408S, and S371F, and T376A and D405N mutations are common with the BA.3 lineage. The newly evolved BA.4 and BA.5 lineages of Omicron are similar to the BA.2 lineage, except for the deletion of an amino acid at positions 69 and 70 and F486V and R493Q mutations in the RBD of the spike protein compared to the BA.1 lineage [9]. The mutation F486V in the spike protein is the leading cause of the infection. The BA.4 and BA.5 sub-lineages have substitution mutations in the RBD: L452R, F486V, and R493Q, compared to BA.2, which does not (Table 1).

The strength of the binding affinity of the RBD region of the Omicron variant to the receptor ACE2 is 1.5–2.8 times higher than that of the wild-type strain [46,47,48,49]. In comparison with the Delta variant, the Omicron RBD part has a weaker or a similar binding affinity to ACE2 [48,49,50,51]. However, the binding affinity of Omicron’s RBD to ACE2 is weaker than that of the Alpha variant. The alpha variant has only one mutation (N501Y) in the RBD region [46,50]. The strength of the binding affinity of the Omicron variant’s RBD to ACE2 is in between those of the RBDs of the wild type and the Delta variant of SARS-CoV-2. The mutations, namely, T478K, S477N, Q496S, Q493R, and Q498R, in addition to N501Y, are thought to potentiate the interaction between the Omicron variant and human ACE2 by forming new salt bridges or hydrogen bonds with the ACE2 receptor [46,48,52,53].

On the other hand, K417N and E484A can cause a loss of interaction between Omicron and the ACE2 receptor part, which the other mutations may have enhanced [46,53,54]. The N501Y mutation in Omicron was also seen in the Gamma, Alpha, and Beta variants, and it augments the binding strength of the spike protein with ACE2. The transmissibility increases further in the N501Y mutation with an added H69/V70 deletion [55,56]. The N679K and P681H mutations incorporate essential amino acids near the furin cleavage site. This facilitates spike protein cleavage in the S1 and S2 subunits, tighter binding, and enhanced virus infectivity [29]. This enhances fusion and virus infection [57]. However, the effects of most of the mutations in the Omicron variant are still unknown. Once all of the roles the mutations play have been identified, the generation of effective vaccines, and thus, the prevention of the disease becomes easier.

## 4. Omicron Variant-Associated Disease Intensity

Omicron shows a disease severity that is lower than that of the other variants, which may be because of its faster growth rate and transmissibility, detrimental changes in the epidemiology of the previous variants, the more virulent nature of the virus or the clinical presentation of the disease, the decreased effectiveness of the vaccines or other therapeutics, or the decreased effectiveness of the social or public health measures. Of all of the Omicron lineages, BA.4 and BA.5 are more transmissible than the others are, which could be because of the higher growth rate of BA.4 and BA.5 than the that of the other sub-lineages. BA.5, which is the most predominant one, was first identified in January, and it was detected by the WHO in April. They can readily evade the immune system, induced by vaccination or viral infections [58]. The original Omicron strain is less severe than the Delta strain is, but the BA.5 variant, along with the BA.4 strain, is most the contagious one, causing more than 50% of the cases due to this variant. All of the Omicron variants, in general, also have a much higher transmissibility rate than the Delta variant does [6]. Omicron is associated with milder symptoms, decreased hospitalization and mortality, and the generation of more asymptomatic carriers compared to infections with other variants [59,60,61,62]. By comparing the Omicron lineages with the other SARS-CoV-2 variants such as the Alpha or Delta ones, the data show that Delta is the most prevalent type in terms of severity. At the same time, Omicron is noted as the most transmissible variant [63]. The effective reproductive number of the Omicron variant (8.2) elicited a 3.8 times higher transmissibility rate than the Delta variant did [15]. The Omicron variant significantly multiplied the number of daily hospitalization cases by three the number of daily cases caused by the Delta variant. However, the number of daily ICU cases was lower in the case of the Omicron variant. The number of everyday hospitalizations during the peak of Omicron was around one time higher in the US and UK than it was during the peak of the Delta variant. This is true for both the minimum and the maximum number of cases. The maximum number of daily ICU cases was similar for the Delta and Omicron variants during the peak outbreak, and the number of daily ICU cases were reduced in every country. During the Delta outbreak, Brazil’s maximum number of deaths was 1857.43 per million. In France, it was 11.86 per million, while in India, it was 3387.71 per million. The number in other countries of interest such as the UK it was 12 per million, and in the US, it was 432.29 per million. During the Omicron outbreak, the maximum numbers of deaths in Brazil, France, India, the UK, and the US are 831.14, 328.86, 1117.71, 86.86, and 2576.71 per million respectively. During the Omicron outbreak, Brazil and India had a lower number of daily deaths than the other countries did [15,64]. Similarly, the degree of the severity of the illness was much lower than it was during the Delta outbreak [65]. The vaccine’s effectiveness also decreased much faster for the Omicron variant than it did for the pre-Omicron variants, and people infected with pre-Omicron variants have only 15% protection against the BA.4 and BA.5 variants.

## 5. Clinical Characteristics and Symptom Prevalence

**Figure 5:**
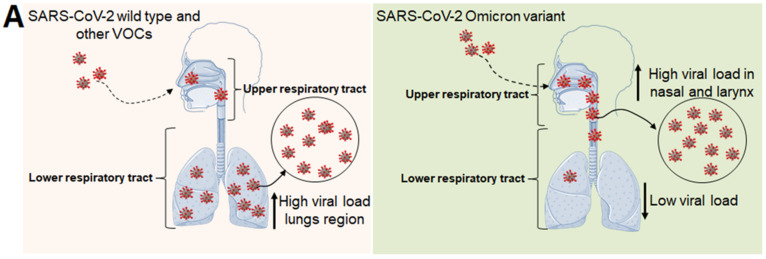

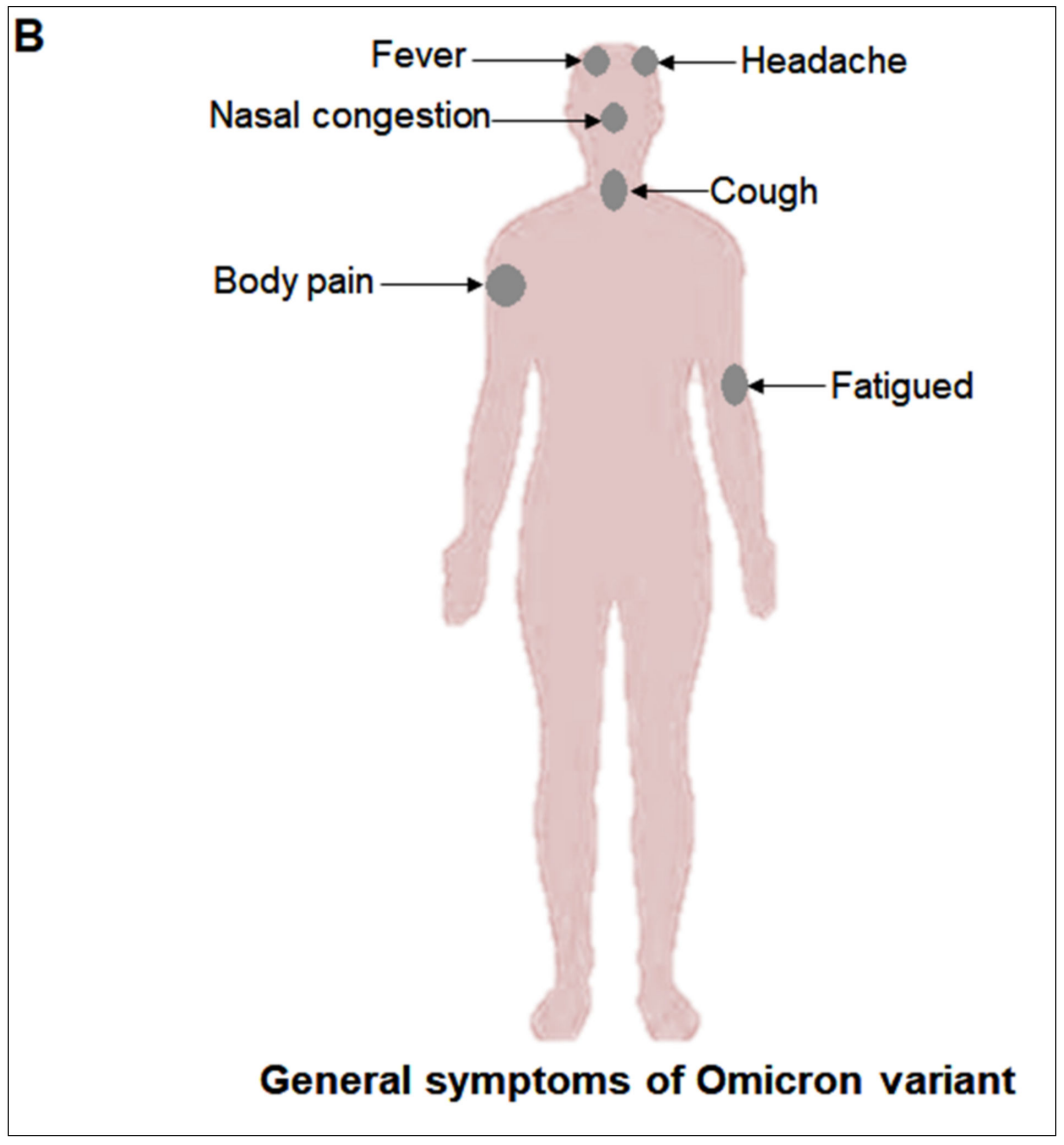
The figure shows viral load in the respiratory tract and the lungs during an infection with the wild strain of SARS-CoV-2 and Omicron. The figure also describes the common symptoms of Omicron-infected patients. (**A**) It shows high viral load in the respiratory tract during Omicron infection. It shows high viral load in lungs during an infection with the wild strain of SARS-CoV-2. (**B**) The figure depicting the common clinical symptoms of Omicron-infected patients.

Several scientists have studied the Omicron variant’s disease intensity and found that it has an increased transmissibility rate and a higher growth rate. However, the Omicron’s higher growth and transmissibility rates might be associated with the viral load. Studies have noted that the viral load is higher in the lungs during the infection of a wild strain of SARS-CoV-2. They also reported that the viral load is higher during an infection with Omicron in the upper airway, especially in the nose, windpipe, and throat, but not in the lower respiratory system [66]. The higher growth and augmented viral load may cause the virus particles to aggregate in the upper airway (Figure 5A).

Omicron is not worse than other coronavirus strains, and it is less severe (less ICU hospitalization) than the Delta variant. The number of individuals with oxygen supports was also smaller than it was during the previous waves due to other SARS-CoV-2 variants, specifically Delta [67]. The clinical characteristics of the SARS-CoV-2 Omicron variant are different from those of all of the previous variants [68,69,70,71]. The most common symptoms are a cough, runny nose, congestion, and fatigue (Figure 5B) [71]. The loss of smell and taste, fever, dizziness, headache, runny nose, hair loss, and blisters on the feet were more adequately prevented during the Delta outbreak than they were during the Omicron outbreak. A sore throat and a hoarse voice were more prevalent during the Omicron outbreak. Individuals infected with Omicron are less likely to show at least one of the three classic symptoms of COVID-19: the loss of smell, a fever, and a persistent cough, which associated with individuals infected with the Delta variant [71]. A study showed that acute symptoms prevailed for a more extended period in patients affected during the Delta outbreak (average of 8.89 days) than they did during Omicron outbreak (average of 6.87 days). It also showed that 1.9% of the vaccinated individuals were admitted to hospitals during the Omicron outbreak compared to 2.6% during the Delta outbreak [71,72,73]. A high number of asymptomatic carriers were identified during the outbreak of the Omicron variant, suggesting that it caused milder symptoms [59]. Respiratory distress is a common symptom in all age groups. Among the gastroenterological symptoms, vomiting is the most common one, and diarrhea and abdominal cramps are common in children aged 5–9 years who are infected with Omicron. Children in the age group of 9–11 show less severe symptoms than infants do, which is valid for both the Delta and the Omicron variants. There have been reports of seizure-associated infections caused by the variant [74]. In vivo studies showed that the Omicron variant did not cause a significant loss of body weight, the viral load was lower, and the amount of lung damage was significantly smaller, and the mortality rates were also lower compared to those of other variants [75,76,77]. Omicron tends to stay in the upper respiratory tract, such as in the nose, throat, and bronchi, rather than settling in the lungs [78,79]. However, in severe cases, pneumonia, respiratory failure, and death can also occur [80,81]. Bronchitis, hypertension, and diabetes are significant comorbidities in individuals infected with the Omicron variant. Another study showed that 36.1% of the Omicron-infected individuals did not show any antibody response, 62.7% of them produced IgG, and 1.2% of them produced IgG and IgM. Many of the Omicron-infected individuals showed abnormally high WBCs, lymphocytes, monocytes, and neutrophils levels, which can lead to monocytosis, neutrophilia, lymphocytopenia, and leukocytosis, while the RBCs and hemoglobin levels were in the normal range [82].

Regarding the transmissibility and effectiveness of the vaccines against the variants, Alpha is 50% more transmissible than the original Wuhan strain is, and it is associated with increased disease severity [83,84,85]. However, the vaccines and monoclonal antibodies remain effective against the variant. The Beta strain is again 50% more transmissible than the previous strains are, but it is not related a more severe disease. It has a reduced neutralization efficiency by the antibodies, and people who have been previously infected are at a greater risk of being reinfected. The Gamma variant is 1.7–2.4 times more transmissible than the non-VOCs are, and patients who have been previously infected with COVID-19 have 54–79% protection against the variant, and the existing vaccines work well against the variant [84]. The Delta variant is 40–60% more transmissible than the Alpha one is, and it is twice as transmissible as the Wuhan strain. Vaccines are less effective against the Delta variant [29,64,85,86]. The vaccines are least effective against the Omicron sub-lineages, especially the BA.4 and BA.5 ones. Another new sub-lineage of Omicron, BA.2.75, which was first found in India in June 2022 is spreading faster than the BA.5 variant did, and it also evades the protection by the immune system caused by a previous infection or antibody generation. However, there are no unique symptoms related to BA.2.75 infection, with a mild fever in most cases, and sometimes, the patients are even asymptomatic. The Omicron variant is the most transmissible one of all the other variants, but the severity of the disease is comparatively lower. As the Omicron variant has mutations that lead to higher transmissibility and better immune escape, the combined mutations are responsible for Omicron’s dominance over the other variants.

The COVID-19 pandemic is a global challenge, and it is necessary to improve healthcare systems, especially the vaccination rates in developing countries. Omicron is highly transmissible, but it is less pathogenic than the other SARS-CoV-2 variants. Even double-dose-vaccinated people with comorbidity are not protected against Omicron [87]. However, immunity can prevent the severity of COVID-19. Increased immunity among the vaccinated population and them having been previously infected can reduce the severity of COVID-19, and SARS-CoV-2 can become endemic, similar to other seasonal viral infections. However, Omicron may still cause severe COVID-19 and death, especially in comorbid and unvaccinated individuals.

## 6. Infection, Reinfection, and Transmissibility

Mutations in viruses is are widespread phenomena. The SARS-CoV-2 virus is not exempt from this. The main question that is speculated by the entire scientific community is the plentiful number of mutations residing in the genome of the Omicron variant, which has significantly decreased the chances of the occurrence of primary infections, but it has resulted in a more significant increase in the chances of reinfecting individuals [88]. It uses the spike glycoprotein, which binds with the host ACE2 receptor and mediates the membrane fusion by utilizing furin and cathepsin L or TMPRSS2 [89]. Most importantly, this variant is more highly contagious than the previously evolved strains are [90]. Similar to the other mutated variants of SARS-CoV-2, Omicron too shares the same procedure of infecting the host cells. The infectivity rate of the Omicron variant is many folds greater than that of the Delta variant [16]. Before November 2021, the rate of reinfection worldwide was considerably low, around 2%, as implied by some international reports. After the emergence of the Omicron variant, the scenario changed. A deeper look at the reinfection rates of Omicron in a place in South Africa elucidated that this variant is more proficient at reinfecting individuals due to its capability of escaping the immune system [91,92,93]. According to the GISAID data, the Omicron variant consists of 11 mutations in the N-terminal domain with an insertion and six deletions. The ins214EPE and N211 mutations present in Omicron have not been reported in any other mutant variants that has evolved before this one [24]. Out of the five VOCs that have been declared to date, some of the mutations responsible for other viral fitness are D614G, T478K, N501Y, and K417N. Besides these mutations, Omicron possesses several more substitutions, which increases the infectivity rate of these variants by many times [94].

The transmission rate of Omicron is approximately 3.2 folds higher than that of the Delta variant, with a doubling time of ~3 days [95,96]. Among the evolving sub-lineages of the Omicron variant, the BA.2 one is found to be more transmissible than the BA.1 sub-lineage is among household contacts [97]. An incident reported in Norway details an alarming scenario about the transmission of the Omicron variant. Of 117 individuals who went to a party, 76% of them were Omicron victims. Out of all of them, 96% of the people who attended the party had been vaccinated. This alarming fact highlights the high transmission rates of this variant, even in the fully vaccinated subjects [98]. Notably, the elevated rates of Omicron transmission can also be due to its potent immune evading capacity, nullifying the vaccinated subjects’ neutralization capabilities [99]. Apart from this, altered cellular tropism and different pathways of infecting host cells may contribute to the increased transmissibility of Omicron [100,101]. The infection landscape of the Omicron variant describes the silent transmission of the virus from one individual to another, as some victims of Omicron rarely show any symptoms [90]. Some of the ancillary factors responsible for Omicron transmission is the binding of the RBD with the hACE2. However, the exact facts about the viral loads after an infection with Omicron remain undiscovered [102,103].

## 7. Omicron Entry and Associated Immunological Features inside the Host Cells

The entry of the SARS-CoV-2 virus inside the host cells is mainly facilitated by the S-glycoprotein [104]. Recent investigations elaborated that this Omicron variant follows an altered cellular entry route. Instead of entering through the plasma membrane, the Omicron variant follows the endosomal entry pathway, which is enhanced by the cathepsins instead of TMPRSS2. Willett et al. also found that pseudotyped Omicron variant infection was more prominent in the cells with a lower expression of TMPRSS2 than it was in those with a high TMPRSS2 expression level. This, in turn, proves the affinity of Omicron’s entry inside the cell through the endosomes [105]. Of several mutations, P681H, N679K, and H655Y reside in the region adjacent to the furin cleavage site. In the case of the previously emerged variants, Gamma and Alpha, the P681H mutation mediates the cleavage [106]. For Omicron, the scenario is slightly different. This variant’s cleavage efficiency is lower than it is for the others, suggesting that the N679K and H655Y mutations impede the cleavage [107]. Unlike the other variants, Omicron possesses additional mutations in all of the structural proteins. The mutations in the N and S proteins escalate the cellular permeability of the Omicron variant.

Additionally, this mutation favors a more robust capsid assembly, which is almost three folds greater than that of the newly emerged Delta variant [108]. The Omicron variant also uses similar protein receptors as the other emerged variants do. Experimental evidence indicates that Omicron entry was predominant in cells with a higher number of ACE2 receptors [22]. Willet et al. elucidated that the changing of Omicron’s preferred entry route indicates that it will have more replication fitness in the upper respiratory tract. Due to the enormous amount of alterations in the spike protein, the recently emerged sub-lineages of Omicron, especially the BA.1 and BA.2 ones, do not form syncytia, which are mainly formed during the initial stages of the processing of the spike protein at the boundary of the two subunits, namely, S1 and S2. These changes, along with the switched entry route, alter cellular tropism [109].

Kared et al. have mentioned the immunological events associated with the entry of Omicron variants inside the host cell, especially for vaccinated individuals. Omicron entry triggers the production of both the cytotoxic and follicular T helper cells, along with a massive surge of RBD and spike-related IgG+ B cells known as plasmablasts, along with some memory B cells [110]. The B cells follow the exact mechanism of neutralization as that which is seen in the wild-type SARS-CoV-2 variant. The B cells derived from the pool of live memory cells has a similar interaction pattern with the S-glycoprotein of Omicron as that of the wild-type variant [111].

## 8. Interaction of Host ACE2 and Capability of Binding with RBD

Compared to Delta, the Omicron variant exhibits an extreme affinity for the ACE2 receptor and accelerates the transmission rate of this variant by many folds. The mutations that result in the extremely high affinity of the Omicron RBD with the human ACE2 receptor are Q493R, T478K, S373P, N501Y, Q498R, S371L, and S375F (Table 1). Additionally, Omicron’s S protein and RBD harbors some amino acids such as leucine and phenylalanine, which are naturally hydrophobic [112]. Some of the mutations in the Omicron variant even contribute to the formation of salt bridges or several hydrogen bonds, which contribute to the binding of the spike protein with hACE2. The polar contacts between Omicron and ACE2 can be significantly lost by K417N and E484A, negating some of the improved interactions created by other mutations [46,51,53].

A deeper look at the crystal structures of the RBD–ACE2 complex of Omicron indicates that the surface area that the Omicron variant can access for interaction with the host is much more prominent than it is for the Delta variant [51]. According to Jung et al., out of the 31 alterations in the spike protein of the Omicron variant in comparison to those in the wild-type variant from Wuhan, 12 changes are found in the S1 subunit of the spike protein, which reside very near the N-terminal region. Fifteen changes can be seen in the receptor-binding domain, with more than five mutations residing near the C terminal. Moreover, the RBD, which forms a direct connection with hACE2 for binding, harbors ten significant mutations, thereby altering the affinity of the spike protein to bind with the host receptor [113]. Among all of the emerging variants, Omicron is highly transmissible. Computational studies regarding the RBD–hACE2 complex of Omicron evidence that it is incredibly stable due to the replacement of some uncharged amino acid residues with lysine and arginine [6]. T478K, Q498R, N440K, and Q493R are some of the mutations present in the RBD of Omicron’s spike protein, where there are some replacements with positively charged residues, thereby improving the binding of RBD to the human ACE2 receptor. Owing to the growth of the side chain, the T478K mutation is situated very close to a solvent-prone area, permitting the interaction between ACE2 and Omicron’s RBD. Furthermore, the Q493R mutation enables an advantageous interaction with certain damaging amino acids such as Glu35 and Asp38 in the ACE2 receptor. It also enables a powerful binding effect [6].

## 9. Phylogenomics and Distribution of Omicron and Its Sub-Variants

**Figure 6:**
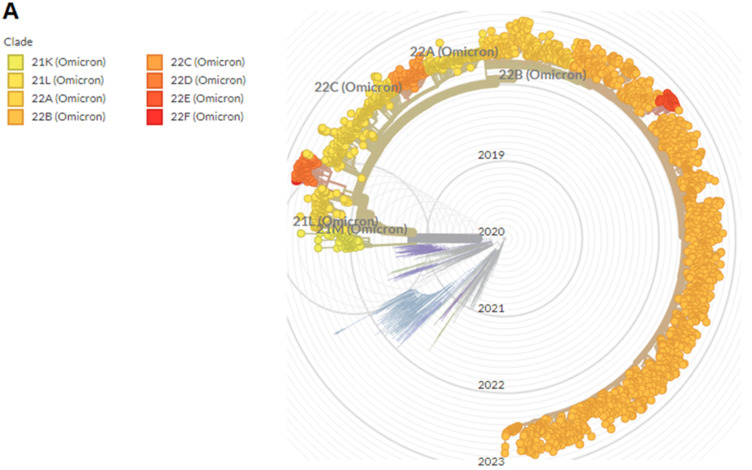

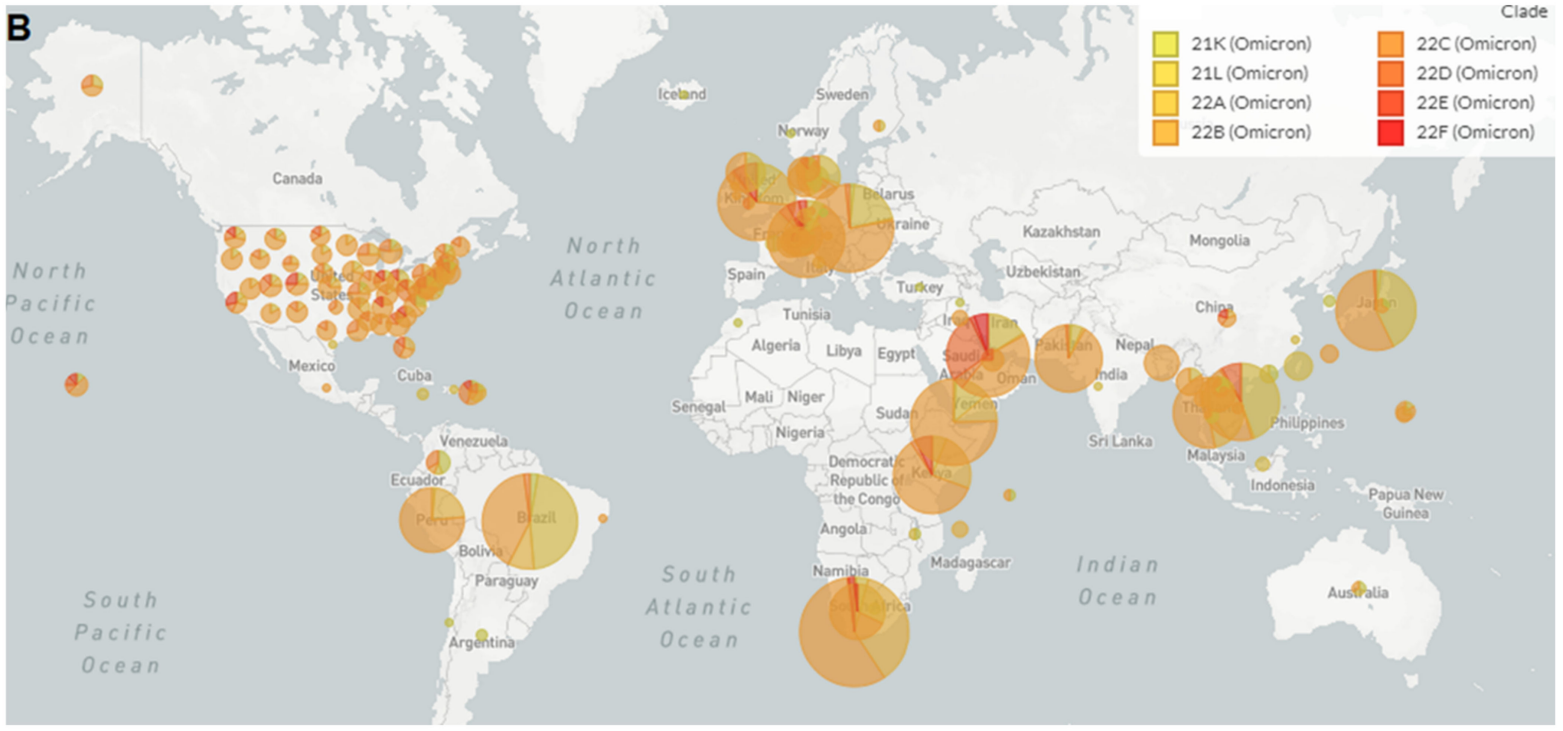
The figure shows the phylogenetic tree of the Omicron and its sub-variants. It also describes the distribution of Omicron and its sub-variants in the entire world. (**A**) The circular phylogenetic tree using the Omicron and its sub-variants (**B**) The distribution of Omicron and its sub-variants. These two figures (**A**,**B**) were developed using the next strain server.

Several scientists have studied the phylogenomics of Omicron and its variants, and their studies have immense importance with respect to the evolution of the virus (Figure 6A). Recently, we have found the phylogenetics of Omicron and its sub-variants. Callebaut et al. described the phylogenetic properties of the BA.1 and BA.2 variants. Samples were collected from Omicron-infected patients [114]. Kandeel M, El-Deeb demonstrated the evolutionary relationships of the RBD of SARS-CoV-2 using a phylogenetic tree. The study placed the Omicron variant into a novel monophyletic class [115]. Additionally, it also described the rapid appearance of multiple sub-variants of Omicron and their divergence [116].

It is also essential to understand the distribution of Omicron and its sub-variants. After the first identification of the Omicron variant in South Africa and Botswana, the variant spread throughout the globe, and several sub-variants, BA.1, BA.2, BA.3, BA.4, and BA.5, generated over time and were spotted throughout the world (Figure 6B). Recently, a new sub-variant, BA.2.75.2, was generated in India, which might be of global concern [20]. We need to obtain more detailed information about the distribution of several sub-variants of Omicron.

## 10. Immune Escape of Emerging Omicron Variant and Its Sub-Variant

In general, vaccine effectiveness against severe diseases is a matter of concern. The vaccine’s effectiveness is not largely affected by the variants. This is because of the mutations of the variants which hinder the neutralization potency of any vaccine. New variants have developed as a result of certain mutations. Several mutations have been observed in the newly developed variants which alter the binding region of nAb, leading to antibody escape [117,118]. In the Omicron case, several mutations have been noted in the nAb binding region of the S protein, especially in RBD and NTD, which cause the nAb escape phenomenon [13,19,27,28,29,119,120]. Therefore, we can say that the Omicron variant possesses a partial vaccine escape ability.

Recent studies elucidate that the Omicron variant and the three sub-lineages, BA.1, BA.2, and BA.3, are very competent in escaping the immune system. The subjects who have taken one or two doses of the vaccine cannot protect against this variant significantly, thus, the neutralization efficiency of these vaccines is gradually decreasing. Most surprisingly, people who had received three shots of the vaccine only have partial protection from the infection of this variant. However, vaccine escape is a common phenomenon. Several researchers urge for the development of new vaccines against the Omicron variant. However, several researchers or pharmacological companies have developed new vaccines against the Omicron variant (Table 2). Similarly, a bivalent COVID-19 vaccine (ancestral and Omicron) can provide long-term protection. Recently, ModernaTX has developed an mRNA-based bivalent Omicron-containing vaccine. The study is now in Phase II and Phase III. The study has evaluated the safety and immunogenicity of the mRNA vaccine boosters (bivalent Omicron-containing vaccine). Chalkias et al. have published data of a clinical trial and evaluated the immunogenicity, reactogenicity, and safety the bivalent Omicron-containing vaccine (mRNA-1273.214). In this study, they assessed three parameters of the bivalent vaccine on the 28th day after the booster dose. Here, the participants received either mRNA-1273 (n = 377) or 50 μg of mRNA-1273.214 (437 participants) as a second booster dose. The researchers found that mRNA-1273.214 (the bivalent Omicron-containing vaccine) elicited superior neutralizing antibody responses compared to the mRNA-1273 vaccine ones against the Omicron variant (ClinicalTrials.gov; Clinical trial: NCT04927065) [121]. Other than the bivalent vaccine of ModernaTX, Pfizer-BioNTech has also developed a bivalent COVID-19 vaccine. A recent clinical trial has been conducted to understand the vaccine’s safety profile (ClinicalTrials.gov; NCT04977479). The first dosage of the mRNA vaccine produced a systemic allergic reactions in some individuals. The researchers want to study the safety profile of giving a second mRNA COVID-19 vaccine to individuals who had developed a systemic allergic reaction to their first dose. Similarly, these two bivalent vaccines’ safety profiles have been assessed in kidney transplant recipients (ClinicalTrials.gov; NCT05518487). It is one of the most likely reasons responsible for the rapid spread of the Omicron variant in countries where people have natural immunity or a rapid vaccination rate [109]. The various mutations in the N-terminal and the RBD of the Omicron variant are the sites that are majorly targeted by the antibodies [122,123,124,125]. The high mutation rates in these positions are the key factors responsible for changing the antigenicity.

Moreover, this antigenic shift can even nullify the overall immunity in the host’s system [126]. The conformation of the S protein and RBD is a significant factor dominating antibody recognition. The trimeric spike complex of the Omicron variant adopts a single “up” conformation, with the RBD keeping the other two in the “down” conformational state, which is a bit different from the previously emerged variants [46,127]. The stearic hindrances induced due to the mutations are solely responsible for altering the interactions in the antibody-binding sites. The unprecedented changes in the spike protein also interfere with the recognition of the antibodies [126,127]. Some of the mutations in the Omicron variant such as Q498R, S477N, Y505H, G496H, and Q493R, along with the other mutational changes prevalent in the VOCs such as T478K, N501Y, and E484K are majorly involved in altering its antigenicity, with this variant being more efficient at escaping the immune system [128]. Cui et al. have mentioned that the two main sites for neutralization, the RBD and the NTD, are heavily mutated in the Omicron variant, which causes severe changes in the conformation of several antigenic sites. The three minor deletions, four substitutions, and one insertion of a 3-residue-long amino acid in the N-terminal region have been the primary cause behind the immune escape strategy of the Omicron variant [46].

Furthermore, the recently reported sub-lineages of Omicron, BA.4, BA.5, and BA.2.12.1, have illustrated more robust strategies for escaping the immune system than the BA.1 and BA.2 sub-lineages have. The BA.1 variant can produce several copies of BA.1-specific antibodies that can be effective against BA.1 infection. However, the other sub-lineages, namely, the BA.4/BA.5 and the BA.2 variants, can invalidate the neutralization efficiency of these antibodies because of the presence of F486V and D405N mutations [37].

## 11. Antiviral Drugs and Antibody-Based Therapeutics against the Omicron and Its Sub-Variants

Several antiviral drugs and antibody-based therapeutics have been investigated and proposed over time against Omicron and its sub-variants. The investigated and proposed antiviral drugs and antibodies are discussed below.

### 11.1. Efficacy of Antiviral Drugs

Numerous antiviral options have been explored for emergency use in hospitalized and non-hospitalized patients to reduce the clinical severity in patients infected with the SARS-CoV-2 wild strain and other variants, including Omicron. A number of antivirals have been proposed against Omicron, such as Remdesivir, Molnupiravir, Camostat, and Ensovibep. These antivirals have been investigated over time to assess their antiviral activities against Omicron and its sub-variants [66]. Takashita et al. have recently evaluated the antiviral activity of three antiviral molecules, such as Remdesivir, Molnupiravir, and Lufotrelvir. In this study, the researchers used three chemicals, namely, 441524, EIDD-1931, and PF-00835231, as therapeutic molecules. The study indicated that these three compounds had efficacy against the Omicron variant. In this study, the researchers evaluated the drugs’ susceptibility to GS-441524, EIDD-1931, and PF-00835231 using a 50% inhibitory concentration (IC50) value. The value for each of them was found to be 1.2, 0.8, and 0.7, respectively. However, the data are different due to the influence of different factors. Here, GS-441524 is an RdRp (RNA-dependent RNA polymerase) inhibitor, and the molecule is the active form of Remdesivir. Similarly, EIDD-1931 is also an RdRp inhibitor, and the molecule is an active form of Molnupiravir. At the same time, the study confirms that Omicron-infected patients can be treated with these drugs. PF-00835231 is a protease inhibitor, which is the active form of PF-07304814 [129]. PF-07304814 is known as Lufotrelvir, which was developed by Pfizer. Similar to remdesivir, this molecule can be administered by intravenous infusion.

Another oral protease inhibitor that has been found by researchers is Nirmatrelvir. Arbel et al. evaluated the activity of these molecules in 109,254 patients. During the study period, 4% of the total number of patients (3902) received Nirmatrelvir. The researchers found that the death and hospitalization rates were significantly lower among the Nirmatrelvir-receiving patients compared to that among the patients who did not receive any dose [130]. The USFDA approved the drug through EUA (an emergency use authorization) for treating of mild-to-moderately infected patients. It was approved for oral use in December 2021. Bojkova et al. have assessed the effects of some molecules, such as Remdesivir, Favipiravir, Ribavirin, EIDD-1931, PF-07321332, Camostat, Nafamostat, and Aprotinin, in Omicron-infected cell cultures. They found similar kinds of antiviral activity among the Delta and Omicron isolates [131]. Vangeel et al. performed an in vitro antiviral assay and reported that Remdesivir (parent nucleoside GS-441524), Molnupiravir (parent nucleoside EIDD-1931), and Nirmatrelvir showed antiviral activity against Omicron. These molecules have also shown antiviral activity against the wild strain of it and other VOCs [132].

The Nirmatrelvir–Ritonavir combination is now an essential antiviral option against the Omicron variant. Several scientists have explained the activity of the Nirmatrelvir–Ritonavir combination against Omicron. Recently, from a cohort study with COVID-19 patients (N = 41,255), Wong et al. stated that the molecules could be considered to be therapeutics for the early phase of the infection [133]. In another study, Wong found that the Nirmatrelvir–Ritonavir combination could have been a therapeutic agent during the early phase of the infection during Omicron BA.2’s wave. The researchers concluded this from a cohort study in Hong Kong [134]. A recent article describes the recommended indications, antiviral activity, pharmacokinetics, mechanisms of action, clinical trial of drug interactions, and adverse affects of the antiviral molecules such as Molnupiravir and the Nirmatrelvir–Ritonavir combination (Paxlovid) against the Omicron variant [135]. Therefore, these two molecules (Molnupiravir and the Nirmatrelvir–Ritonavir combination) are significant additions to the early phase of the treatment of COVID-19, especially to Omicron and its sub-variants.

### 11.2. Efficiency Therapeutic Antibodies

Scientists are facing a real challenge to finding therapeutic antibodies against the Omicron variant because the therapeutic antibody escapes their neutralization efficacy due to certain properties of the Omicron variant [120,136]. Due to this, several scientists have tried to evaluate therapeutic antibodies against the Omicron variant over time and assess their efficacy of naturalization (Table 3). Recently Tao et al. published a meta-analysis and systematic review, where they found that several studies were involved in understanding the susceptibility of mAbs (monoclonal antibodies) against the Omicron variants [132]. Takashita et al. have assessed the antibodies against Omicron which are Bamlanivimab (LY-CoV555), Imdevimab (REGN10987), Casirivimab (REGN10933), Tixagevimab (COV2-2196), Cilgavimab (COV2-2130), and Sotrovimab precursors (S309). The researchers also evaluated a plethora of the combinations of monoclonal antibodies. Some of the combinations include Tixagevimab with Cilgavimab, Imdevimab with Casirivimab, and Etesevimab with Bamlanivimab. It was also found that these combinations of monoclonal antibodies could neutralize the wild strain as well as the Delta and Alpha variants. At the same time, the combined treatment of Bamlanivimab and Etesevimab highlighted a reduced neutralizing activity against the Gamma variant. Furthermore, these combinations have completely lost their neutralization efficacy against the Beta and Omicron variants [129].

**Figure 7:**
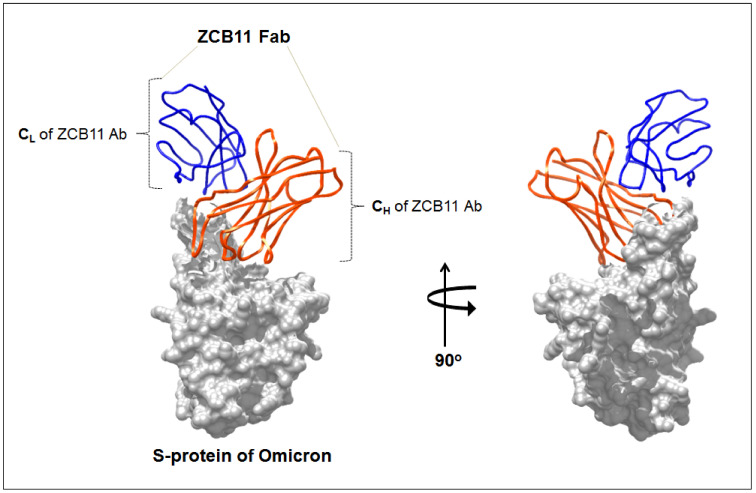
The figure shows the interaction structure of neutralizing antibodies (nAb) with the Omicron spike protein. It shows the interaction Fab fragment of ZCB11 against the SARS-CoV-2 Omicron spike. The structure was developed from a PDB file (PDB id: 7XH8).

Similarly, they also found that the Casirivimab and Imdevimab combination has shown activity against the Gamma and Beta variants. However, this combination failed to neutralize the Omicron one. However, it has been noted that the Cilgavimab–tixagevimab combination has shown significant neutralization potency against the Beta, Gamma, and Omicron ones [129]. Similarly, Tada et al. found from a study that Sotrovimab and Evusheld were partially effective against the Omicron pseudotype. On the other hand, Eli Lilly and Regeneron monoclonal antibodies were found to be ineffective against the Omicron pseudotype [137]. Several in silico studies have been performed to identify the therapeutic antibodies against Omicron. In this field, Shah and Woo have suggested that a cocktail of sotrovimab (GSK, S203 mAb) and Evusheld (AstraZeneca mAbs) could successfully neutralize the Omicron variant [138]. Researchers have also tried to understand the interaction between the neutralizing antibodies (nAB) and Omicron’s spike protein. It might provide a deeper understanding of the specific interaction mechanisms possessed by these antibodies. A recent study informed us that ZCB11 is a promising antibody against the Omicron variant. Zhou et al. have elucidated the interaction between ZCB11 and the spike protein of the Omicron variant (PDB id: 7XH8). The study informed us that ZCB11 targets the viral RBD and neutralizes the spike protein of the SARS-CoV-2 variants such as Delta or Omicron [139] (Figure 7).

## 12. Conclusions

Current data have informed us of the three significant properties of the Omicron variant. Firstly, the Omicron variant causes less severe infections. Secondly, the variant has a very high rate of transmissibility compared to that of other VOCs. Lastly, the Omicron variant has a high immune escape capacity and partial vaccine escape ability. Efforts are being made over time to develop the next-generation vaccine and mutation-proof vaccines [28,140,141]. At the same time, it has been observed that the Omicron variant and its sub-variant possess a very high number of mutations [27,29,120]. These mutations provide three significant properties to the Omicron variant.

Recently, it has been seen that hybrid immunity significantly provide more immune protective against SARS-CoV-2 and the other VOCs [141,142]. Therefore, we need to explore the possibility of hybrid immunity for protection against Omicron. At the same time, researchers have informed us that the Omicron variant might be a possible vaccine candidate. The viral strain can be used as a promising live-attenuated vaccine candidate. Therefore, a strategy has been proposed to find a possible solution to provide protective immunity against Omicron, which is known as the “virus against the virus” [143]. However, a bivalent Omicron-containing vaccine has recently been developed by ModernaTX, which can provide long-term protection against the Omicron variant [121]. Molnupiravir and the Nirmatrelvir–Ritonavir combination (Paxlovid) have been found to be effective therapeutic antiviral molecules against the Omicron variant and its sub-variants. However, further studies are needed on the Omicron variant to obtain a clear idea about its pathophysiology and the infection landscape, which will be beneficial for the development of suitable therapeutics.

## Figures and Tables

**Table 1:** Omicron and its variants have several unique mutations in the RBD region. The RBD mutations might control the functionality of the RBD region.

Sl. No	Omicron Sub-Variant Name	Mutations in S Protein	
		RBD Region	Other Than RBD Region
1.	BA.1	G339D, S373P, S375F, K417N, N440K, G446S, S477N, T478K, E484A, Q493R, G496S, Q498R, N501Y, Y505H	A67V, HV69-, T95I, G142D, VYY143-, NL211I, 215EPE, T547K, D614G, H655Y, N679K, P681H, N764K, D796Y, N856K, Q954H, N969K, L981F
2.	BA.2	G339D, S373P, S375F, T376A, D405N, R408S, K417N, N440K, G446S, S477N, T478K, E484A, Q493R, Q498R, N501Y, Y505H	T19I, LPP24-26-/A27S, G142D, V213G, D614G, H655Y, N679K, P681H, N764K, D796Y, Q954H, N969K
3.	BA.3	G339D, S373P, S375F, D405N, K417N, N440K, G446S, S477N, T478K, E484A, Q493R, Q498R, N501Y, Y505H	A67V, HV69-, T95I, G142D, VYY143-, NL211I, D614G, H655Y, N679K, P681H, N764K, D796Y, Q954H, N969K
4.	BA.4	G339D, S371F, S373P, S375F, T376A, D405A, R408S, K417N, N440K, L452R, S477N, T478K, E484A, F486V, Q498R, N501Y, Y505H	T19I, L24_P26del, A27S, H69_V70del, G142D, V213G, D614G, H655Y, N679K, P681H, N764K, D796Y, Q954H, N969K
5.	BA.5	G339, S371F, S373P, S375F, T376A, D405A, R408S, K417N, N440K, L452Q, S477N, T478K, E484A, Q493R, Q498R, N501Y, Y505H	T19I, L24_P26del, A27S, G142D, V213G, D614G, H655Y, N679K, P681H, S704L, N764K, D796Y, Q954H, N969K

**Table 2:** Vaccines against Omicron in the clinical trial.

SL. NO.	Vaccine	Country of Origin	Company Name	Clinical Trial Number	Phase	Recruitment Status	Number of Participants	Remark
1.	ABO1009-DP vaccine	China	Suzhou Abogen Biosciences Co., Ltd.	NCT05433194	Phase I	Not Yet recruiting	48	A clinical trial which aimed to monitor the safety and efficacy profile of this vaccine against Omicron in fully vaccinated subjects below 18 years
2.	Inactivated Omicron COVID-19 vaccine (Vero Cell) Inactivated	China	China National Biotec Group Company Limited	NCT05365724	Phase II	Recruiting	280	A non-randomized trial which aims to monitor the safety and efficacy profiles of the vaccine in non-vaccinated subjects below 18 years old
3.	mRNA-1273.214 Vaccine	Israel	Sheba Medical Center	NCT05383560	Phase II	Not Yet recruiting	150	A placebo controlled study aimed to evaluate the immunogenicity of Omicron-matched booster doses in adult subjects
4.	SCTV01E	China	Sinocelltech Ltd.	NCT05308576	Phase III	Not Yet recruiting	12,000	A randomized study which monitored the safety profile of SCTV01E in subjects aging 12 years or older
5.	BIBP Omicron Inactivated COVID-19 vaccine	Hong Kong	China National Biotec Group Company Limited	NCT05382871	Phase III	Recruiting	1800	A randomized study which monitors the safety and efficacy of this vaccine in subjects who previously received two or three doses of any vaccine
6.	mRNA-1273.214 (bivalent Omicron-containing vaccine)	United States	ModernaTX, Inc.	NCT04927065	Phase III	Active	5158	Immunogenicity and safety evaluation of bivalent mRNA vaccine boosters for SARS-CoV-2 variants
7.	Pfizer-BioNTech bivalent (Omicron-specific) vaccine	Australia	Murdoch Childrens Research Institute	NCT05543356	Phase III	Withdrawn	1143	Evaluation of bivalent Omicron-specific COVID-19 vaccine booster dose (Pfizer-BioNTech) in healthy adults
8.	Pfizer-BioNTech COVID-19 bivalent vaccine	United States	National Institute of Allergy and Infectious Diseases (NIAID)	NCT04977479	Phase II	Active	17	Safety analysis of the COVID-19 mRNA vaccine (2nd dose) to individuals who had a systemic allergic reaction to a prior dose
9.	Bivalent booster of mRNA based COVID-19 vaccine	United States	National Institute of Allergy and Infectious Diseases (NIAID)	NCT05518487	Phase II	Not Yet recruiting	80	Safety and immunogenicity study of single dose of bivalent (mRNA-based) vaccine to individuals (kidney transplant recipient) with a persistently low SARS CoV-2 antibody titer
10.	Bivalent mRNA COVID-19 vaccine	United States	National Institute of Allergy and Infectious Diseases (NIAID)	NCT05077254	Phase II	Recruiting	400	Evaluation of Ab response to an extra dose of bivalent (mRNA-based) COVID-19 vaccination in subject of immunosuppression reduction in organ (kidney and liver) transplant recipients

**Table 3:** The efficiency of the antibodies effective against Omicron and its sub-variants.

Sl. No.	Therapeutic Antibodies	Neutralization Efficacy in Different Omicron Sub-Variants				
		BA.1	BA.2	BA.3	BA.4	BA.5
1.	Tixagevimab	Low	Low	Low	Low	Low
2.	Bamlanivimab	Low	Low	Low	Low	Low
3.	Imdevimab	Low	Moderate	Low	Moderate	Moderate
4.	Regdanvimab	Low	Low	-	-	-
5.	Sotrovimab	Moderate	Moderate	Moderate	Moderate	Moderate
6.	Casirivimab	Low	Low	Low	Low	Low
7.	Cilgavimab	Low	High	High	High	High
8.	Etesevimab	Low	Low	Low	Low	Low
9.	Bebtelovimab	High	High	High	High	High
10.	Bamlanivimab + Etesevimab	Low	Low	Low	Low	Low

## Data Availability

No new data were created or analyzed in this study. Data sharing is not applicable to this article.
